# A Quantitative Method to Guide the Integration of Textile Inductive Electrodes in Automotive Applications for Respiratory Monitoring

**DOI:** 10.3390/s24237483

**Published:** 2024-11-23

**Authors:** James Elber Duverger, Victor Bellemin, Patricia Forcier, Justine Decaens, Ghyslain Gagnon, Alireza Saidi

**Affiliations:** 1 Institut de Recherche Robert-Sauvé en Santé et en Sécurité du Travail, Montréal, QC H3A 3C2, Canada; james.elber-duverger@irsst.qc.ca; 2 Department of Electrical Engineering, École de Technologie Supérieure, Université du Québec, Montréal, QC H3C 1K3, Canada; victor.bellemin.1@ens.etsmtl.ca (V.B.); ghyslain.gagnon@etsmtl.ca (G.G.); 3 CTT Group, Saint-Hyacinthe, QC J2S 1H9, Canada; pforcier@gcttg.com (P.F.); jdecaens@gcttg.com (J.D.)

**Keywords:** respiratory monitoring, breathing rate, breathing sensor, textile inductive electrode, respiratory signal, signal quality, automobile, automotive applications, seat back, seat belt

## Abstract

Induction-based breathing sensors in automobiles enable unobtrusive respiratory rate monitoring as an indicator of a driver’s alertness and health. This paper introduces a quantitative method based on signal quality to guide the integration of textile inductive electrodes in automotive applications. A case study with a simplified setup illustrated the ability of the method to successfully provide basic design rules about where and how to integrate the electrodes on seat belts and seat backs to gather good quality respiratory signals in an automobile. The best signals came from the subject’s waist, then from the chest, then from the upper back, and finally from the lower back. Furthermore, folding the electrodes before their integration on a seat back improves the signal quality for both the upper and lower back. This analysis provided guidelines with three design rules to increase the chance of acquiring good quality signals: (1) use a multi-electrode acquisition approach, (2) place the electrodes in locations that maximize breathing-induced body displacement, and (3) use a mechanical amplifying method such as folding the electrodes in locations with little potential for breathing-induced displacement.

## 1. Introduction

Within automobile technology innovation, the trend towards contactless vital-sign-monitoring technologies has attracted considerable interest through ongoing research and development efforts to enhance the safety of autonomous driving and facilitate real-time driver health assessments [1,2]. In fact, systems for monitoring the driver’s status are essential for disruptive innovation in autonomous driving. This is particularly important at certain levels of autonomy, where the automobile is responsible for partial or total driving, but the driver must be prepared to take back control if needed. In these circumstances, monitoring the driver’s status makes sure that the driver is alert and ready to take some actions [3,4]. On the other hand, there is a growing demand for integrated systems that can discreetly monitor drivers’ vital signs, with the aim of preventing health-related incidents and reduce road injuries and fatalities [1,5]. As traditional methods of physiological monitoring, including intrusive direct skin contact, are often inconvenient for continuous application in a driving context, contactless systems that monitor physiological signs, such as ballistocardiography, radar, optical vibrocardiography, magnetic induction, capacitive ECG, ultrasonic and infrared sensing, thermal or visible light imaging, and heart rate extraction from speech, have been explored in recent years [1,6,7].

Among the various detection methods, inductive coupling detection is of great interest for detecting respiratory activity. Inductive monitoring methods use coils that produce an alternating magnetic field, which interacts with the thoracic tissue to induce eddy currents. These currents create a secondary magnetic field that opposes the initial one, thereby modifying the coil’s effective impedance, also known as the reflected impedance. When the thoracic conductivity changes due to movements of the thorax and internal organs, the reflected impedance changes accordingly [8,9].

In previous studies, both single-coil setups driven by an oscillator (Colpitts or LC), and multi-coil configurations such as a gradiometer [8,9,10], have been investigated for specific applications, with outcomes that could contribute to the research on inductive respiration monitoring in automotive applications. For example, a single flexible coil embedded in a foam mattress [11] and a multiple-coil configuration in a bed-like structure [12] have been proposed to detect respiration events. Pertaining to wearable inductive respiratory monitoring systems [13], a single-coil system as part of a Colpitts oscillator inserted into a belt behind the left lung [14], circular coils of diverse shapes integrated into the front panel of a shirt [15], a planar flat coil inserted into a clothing chest panel [16], and a low-power wearable inductive monitoring system with 3D-knitted helical coils [17] have been studied over the last few years.

Regarding respiratory monitoring in automotive applications, one study successfully detected respiration rate by integrating a single coil inside the seat back of a car seat [9]. The authors observed that a coil fixed inside the car seat, resulting in a large distance between the coil and the driver’s body, affected the signal quality, especially for shallow breathing—they recommended decreasing the body–coil distance to improve detection. In another study, a single coil allowed reliable respiratory monitoring by being integrated into the backrest of a chair, closer to the body but behind rigid acrylic glass [8]. This result can be extended to automotive applications. However, a rigid seat back in an automobile may be impractical, and the positioning of electrodes for the optimal detection of breathing has not been thoroughly investigated. Beyond the seat back, a single coil embroidered directly into the seat belt to cover the sternum region demonstrated that breathing detection was feasible [18]. Some studies went further, by covering respiratory signal acquisition in driving simulations. For example, a multiple-coil system—with one emitting coil and two receiving coils, implementing a gradiometer—was fixed into a block of resin and then integrated into the backrest of a car seat [10]. The system demonstrated successful respiratory activity with a 5% average error compared to a reference signal. Finally, a recent study validated a design for a magnetic induction sensor for discreet respiration monitoring in an in-car environment involving several hours of road driving and various subjects while achieving an effective coverage rate. The monitoring system, which was mounted on the backrest of the driver’s seat, was based on a Colpitts oscillator including a 20 cm coil of 50 windings [19].

As documented above, respiratory monitoring in automotive applications using inductive technologies has not been studied much in the literature. The use of textile inductive electrodes is even less covered, despite them being interesting candidates to allow seamless integration into seat backs and seat belts, ensuring optimal proximity to the driver’s body. Respiratory events like drowsiness are important in automotive applications and can be detected by the driver’s breathing rate [20], but most reliable inductive technologies for breathing rate detection are used in wearable applications or in hospital environments [21]. Furthermore, earlier studies did not offer effective solutions and guidelines for practical integration of a respiratory monitoring system into an automobile’s operational environment.

In this paper, we introduce a quantitative method based on a signal quality metric to guide the integration of textile inductive electrodes in automotive applications. We illustrate the method in a case study with one subject and a simplified setup. The metric is successfully used to provide basic design rules about where and how to integrate the electrodes to gather good quality respiratory signals in an automobile. More specifically, this study includes the following:

1. Builds a breathing sensing system with textile inductive electrodes and dedicated circuitry and acquisition software;
2. Assembles several realistic design configurations by integrating various numbers of electrodes into diverse places on the seat back or seat belt;
3. Creates and uses a metric to quantitatively assess the signal quality of inductive respiratory signals;
4. Uses a ranking method based on the signal quality to evaluate the design configurations;
5. Deduces basic design rules from the evaluation of design configurations.

Compared to previous works on inductive respiratory signals, our study stands out on four points. First, we specifically address electrode integration in terms of the number, the location on the seat belt versus the seat back, and the geometric disposition of the electrodes. Second, we use noise profiling instead of breathing rate accuracy to allow an effective comparison between the electrodes, even when breathing accuracy is similar. Third, we took an industrial approach to prototyping and the integration of the inductive textile electrodes to test the metric using a realistic way of acquiring signals in an automobile. Finally, we varied the electrode locations, thoroughly covering all areas of the seat back and seat belt.

## 2. Materials and Methods

### 2.1. Sensing Principle and Implementation

**Figure 1:**
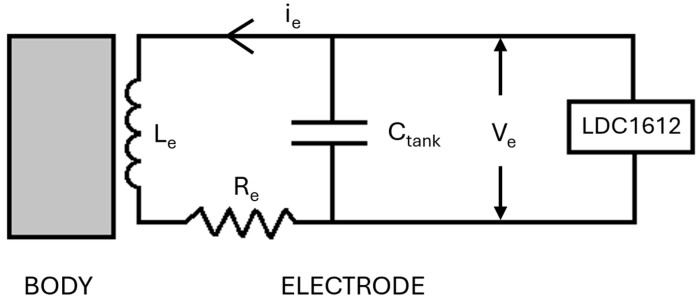
Sensing principle’s equivalent circuit. L_e_ and R_e_ represent the electrode’s inductance and lossy resistance, respectively. The capacitor C_tank_, in parallel with L_e_, creates an LC tank oscillator. The LDC1612 inductive sensing chip drives the LC tank with a current, i_e_, and measures the frequency of the voltage, V_e_.

In this study, we use a textile inductive electrode to sense breathing-induced body displacements. The electrode transduces body displacements into measurable inductance variations. The sensing principle is illustrated by the equivalent circuit in Figure 1. A LDC1612 inductive sensing chip (by Texas Instrument, Dallas, USA) generates the current, i_e_, driving a LC tank oscillator, represented by the electrode’s inductance, L_e_, in parallel with the capacitance, C_tank_. Mechanical interactions between the subject’s body and the electrode changes L_e_, which is indirectly measured by the LDC as the frequency of the oscillating voltage, V_e_, across the LC tank. The series resistance, R_e_, represents the lossy element of the electrode.

With f_e_ being the frequency of the oscillating voltage, V_e_, the value of L_e_ is given by the following formula:

(1) $$L_{e}=\frac{1}{{\left(2\pi \cdot f_{e}\right)}^{2}\cdot C_{tank}}$$

The value of C_tank_ depends on the electrode’s design and will be provided in Section 2.2.1 with further explanations.

**Figure 2:**
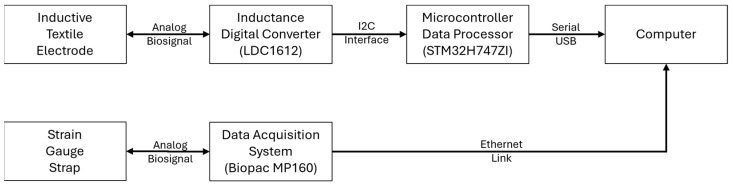
Diagram showing the implementation of the sensing principle. The inductive textile electrode generates an analog respiratory signal that is digitized by the LDC1612 inductive sensing chip, then buffered by the STM32 data processing chip, and finally transferred to the computer. A strain gauge strap around the chest generates a reference analog respiratory signal that is digitized by the Biopac data acquisition system and then transferred to the computer.

The sensing principle is implemented as illustrated in the diagram in Figure 2. The LDC1612 inductive sensing chip transduces the electrode inductance into frequency values (sampled at 40 Hz) that are transferred to an STM32H747ZI data processing chip through an I2C interface. The STM32 chip buffers the frequency values in the internal DMA memory and then transfers them to the computer through a serial USB interface.

A strain gauge strap around the chest transduces the breathing-induced rib cage displacement into an analog signal. A Biopac MP160 data acquisition system measures the strain and transfers the values to the computer via an Ethernet link.

Lab Streaming Layer software (version 1.16.2) synchronizes the data coming from the STM32 and the Biopac. The synchronized data is saved in an xdf file for further processing.

The sampling rate is bounded within the interval [1.834 Hz, 312.5 MHz] by two criteria. At the lower end of the interval, the Nyquist criterium recommends to sample data at twice the highest useful frequency of the signal: 2 × 0.917 Hz = 1.834 Hz, with 0.917 Hz corresponding to the maximum breathing rate of 55 breaths/min considered in this study. At the higher end of the interval, according to the LDC1612 datasheet [22], the sampling rate (called the “conversion interval”) is inversely proportional to the measurement resolution. The highest sampling rate is 1/3.2 µs = 312.5 MHz, with 3.2 µs being the lowest conversion interval. The choice of the sampling rate is free, as long as it is within the interval [1.834 Hz, 312.5 MHz]. The chosen sampling rate of 40 Hz is high enough to analyze the useful frequencies of the respiratory signal and low enough to ensure good measurement resolution.

### 2.2. Electrode Design and Prototyping

#### 2.2.1. Electrode Design

**Figure 3:**
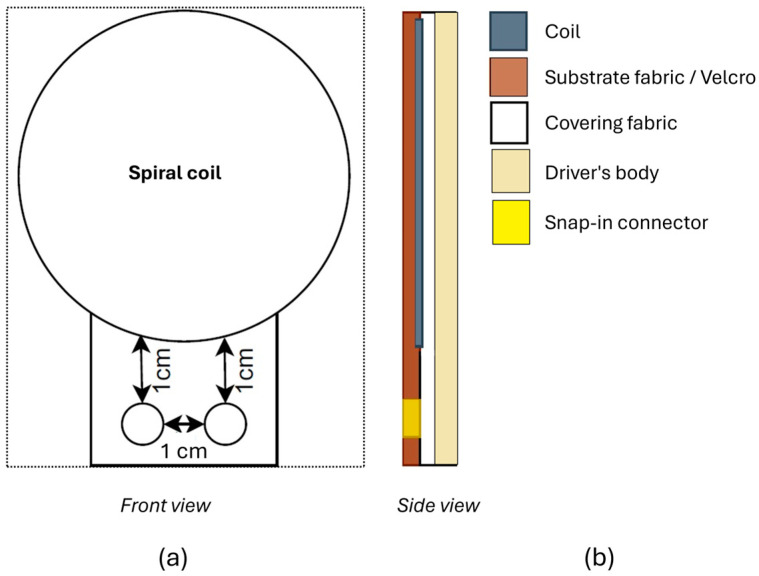
Schematic of the circular electrode design. (**a**) Front view of the circular electrode. It shows the spiral coil (big circle) and the two connectors (little circles) enclosed in the substrate fabric (dotted rectangle). (**b**) Side view of the circular electrode. It shows the spiral coil (in gray) enclosed in the substrate fabric (in brown) and the covering fabric (in white). The snap-in connectors (in yellow) link the electrode to the acquisition circuit.

**Figure 4:**
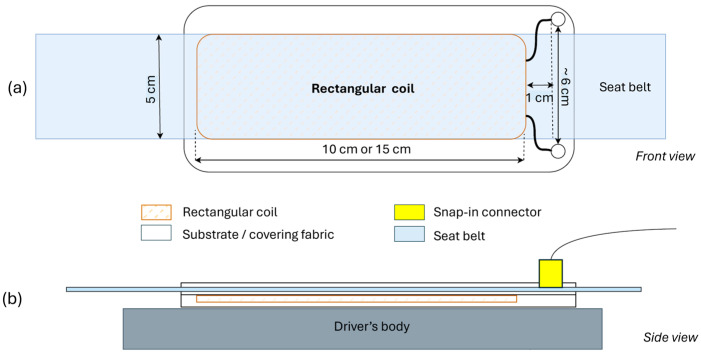
Schematic of the rectangular electrode design. (**a**) Front view of the rectangular electrode. It shows the rectangular coil (brown, dashed rectangle) and the connectors (two little circles) enclosed in the substrate fabric (big, rounded rectangle). The substrate fabric is wrapped around the seat belt (in sky blue). (**b**) Side view of the rectangular electrode. It shows the rectangular coil enclosed in the substrate and covering fabrics (in white). The substrate fabric is wrapped around the seat belt (in sky blue). The snap-in connector (in yellow) links the electrode to the acquisition circuit.

We worked on the design of two types of inductive electrodes to integrate into a seat back or a seat belt, namely flat spiral coils and planar rectangular spiral coils. The calculation of parameters such as the outer/inner diameter, the number of turns, the space between turns, and the yarn diameter were inspired from principles outlined by [23,24] for spiral and rectangular coils, respectively. The schematics in Figure 3 and Figure 4 respectively show the designs for the circular and rectangular electrodes. The main design objective was easiness of integration into the seat back and the seat belt in an automobile’s operational environment.

A total of 5 different designs of inductive electrodes (3 circular and 2 rectangular) were used. as follows:

- A.73.12 with a circular design for integration into the seat belt.
- A.73.15 and A.73.20 with a circular design for integration into the seat back.
- A.73.39 and A.73.40 with a rectangular design for integration into the seat belt.

Table 1 shows the characteristics of each type of electrode. With inductances ranging from 1.7 μH to 19.8 μH, C_tank_ was chosen at 1 nF to keep the LC tank oscillating frequency between 1.13 MHz and 3.86 MHz, within the operating range of the LDC1612 (1 kHz to 10 MHz).

#### 2.2.2. Electrode Prototyping

Our textile inductive electrode is a coil embroidered onto fabric with VELCRO^®^ (by Velcro USA Inc, Manchester, NH, USA) in its contour, enabling it to be integrated into the surface of the seat back or seat belt. Since seat covers and seat belts are typically made from woven fabrics, embroidery emerges as the preferred technique because it allows electrodes to be integrated into the finished material with only one extra manufacturing step added after the fabric is produced. Thereby, the sensors can be seamlessly integrated into the cover or seat belt.

Electrode prototyping was done by the CTT group (Saint-Hyacinthe, Quebec, Canada), which contributed to the project as a scientific partner. To design the electrodes, a Tajima TMLX-1201 embroidery machine running a TFP—tailored fiber placement—process yarn deposition head was used. Embroidered samples were tailored using silver-plated, tin–copper alloy polyamide yarn, CE3286YA, from Maeden Innovation Co., Ltd., Taipei City, Taiwan. This yarn, with a 2-wrap × 4 × 4 strand structure, exhibits a resistivity of less than 0.28 Ω·m. This compound yarn is one of the top choices for embroidering conductive textiles, as it offers the appropriate balance of electrical performance, mechanical strength (breaking load> 17 kg, >50,000 s-rate cycles in a bending test at 270 degrees), flexibility, operational ease, and durability. The silver-plated, tin–copper alloy polyamide yarn combines the high conductivity of silver and copper with the protection of tin for extended reliability. The flexible polyamide ensures ease of handling, while the smooth silver-plated surface of the yarn prevents snagging. In addition to the antimicrobial properties offered by silver and copper, the enhanced solderability, enabled by the presence of tin, makes this compound yarn a superior choice compared to other solutions, such as stainless steel, pure silver-coated, conductive polymer-based, carbon-based, or copper yarns [25,26].

The zig-zag stitch length for the inductive electrodes is 3.0 mm for those with a rectangular shape. For the circular electrodes, the stitch length varies between 1.0 mm for the central spirals and 2.0 mm for the external spirals. The zig-zag stitch is rather tight, so as to stabilize the conductive yarn and prevent any gaps between stitches that could lead to distortion. A black 27 Tex 100% polyester yarn was also used to stabilize the embroidered electrodes on a black substrate, water-repellent poplin fabric, made of 65% polyester/35% cotton and weighing 180 g/m². The same polyester/cotton fabric was used to cover the electrodes to avoid direct contact with the subject’s body. Furthermore, the textile electrodes were connected to the downstream electrical circuit by a stainless-steel snap-in connector for improved mechanical and electrical stability. The stud part of the connector was on the electrode’s side and the socket part on the wire linking it to the circuit.

**Figure 5:**
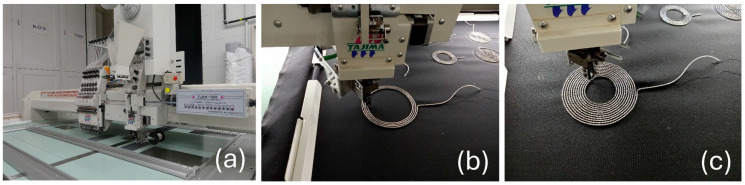
Electrode prototyping. (**a**) Tajima TMLX-1201 embroidery machine. (**b**,**c**) Close-up of the yarn deposition process.

**Figure 6:**
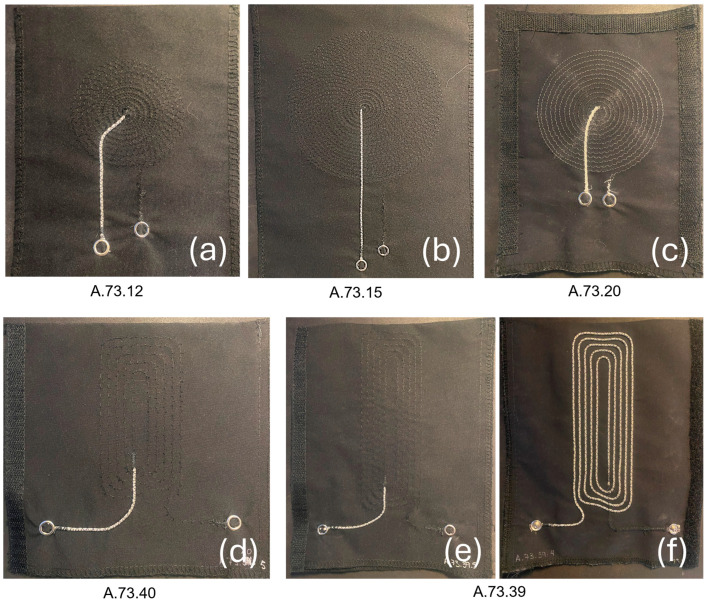
Samples of electrodes: (**a**–**c**) show the circular designs, (**d**,**e**) show the rectangular designs, (**f**) shows a rectangular design on the yarn side.

Figure 5 and Figure 6 illustrate the prototyping process and the samples of electrodes used in this study.

### 2.3. Experimental Setup

#### 2.3.1. Integration of the Textile Inductive Electrodes onto a Driving Seat

**Figure 7:**
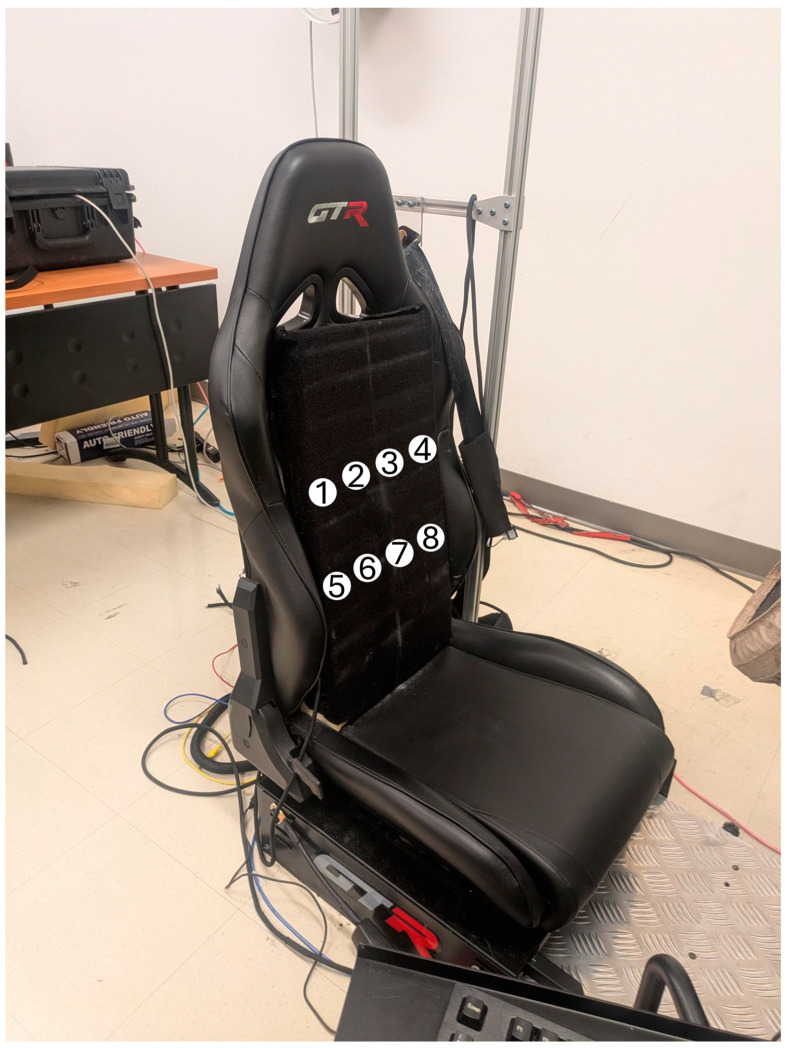
Driving seat and electrode positioning on the seat back. The S105L-BKRD simulator racing seat. A foam covered with loop fabric is used as the seat back. The numbers show approximately where the circular electrodes are attached to the loop fabric during the signal acquisition.

An S105L-BKRD Simulator Racing Seat (Figure 7) by GTR, Ontario, Canada, including a racing belt and 4-way adjustability, was used for the tests.

Polyester-based woven hook and loop VELCRO^®^ was fixed onto the contour of the substrate containing the electrodes for easy placement of the circular electrodes onto the seat back.

A firm 1 cm thick polyurethane foam covered with VELCRO^®^ brand knitted polyester-based loop fabrics was placed on the back of the seat, allowing the electrodes to be attached. The foam also ensured better contact with the subject’s body.

**Figure 8:**
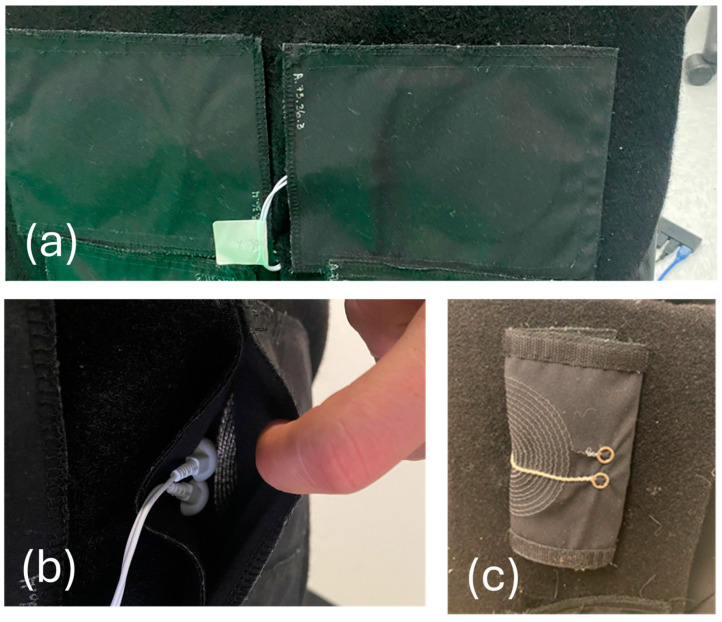
Integration of the electrodes onto the seat back. (**a**) Close-up of the circular electrodes attached to the seat back. The covering fabric hides the spiral coils. (**b**) The covering fabric is lifted to expose the spiral coil and the two connectors. (**c**) The electrode can be folded and then attached to the seat back.

Like the recent method proposed by [27] for folded paper-based inductive electrodes, which demonstrated higher sensitivity in the folding deformation mode due to significant changes in the spatial distance, resulting in a larger variation in inductance for the same displacement, we also conducted some of our experiments on the seat back, with our textile-based electrodes folded. See Figure 8 for an illustration.

#### 2.3.2. Integration of the Textile Inductive Electrodes into the Seat Belt

**Figure 9:**
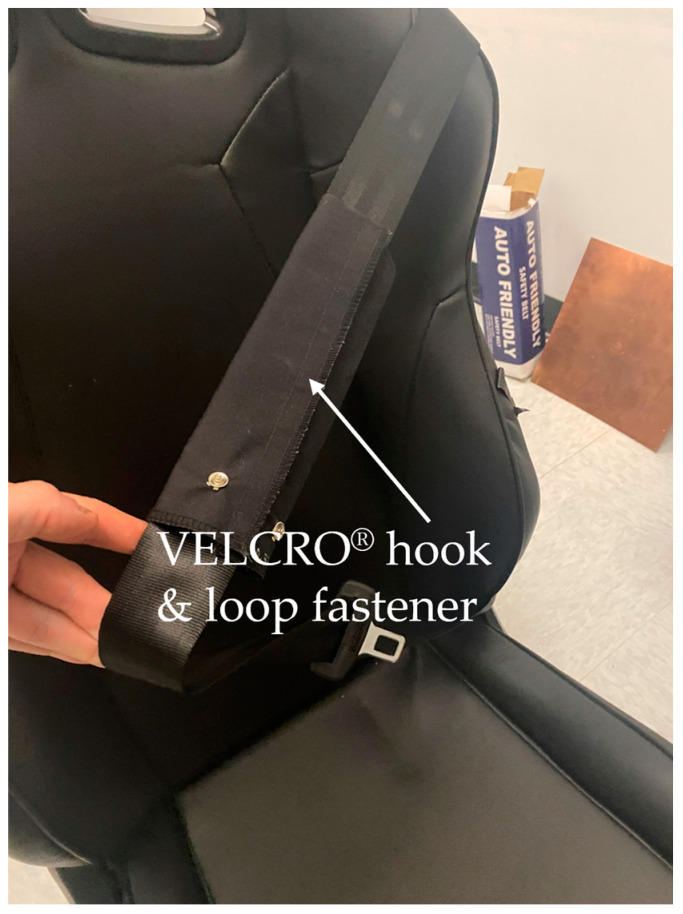
Integration of the electrodes on the seat belt. The electrode is wrapped around the belt with a VELCRO^®^ hook and loop fastener.

The VELCRO^®^ hook and loop fastener also enabled the rectangular electrodes to be attached like a band to the seat belt, by wrapping it around so as the edges of the VELCRO^®^ electrode support fabrics met (see Figure 9).

### 2.4. Signal Acquisition Protocol

Signal acquisition was performed on one male subject in his twenties, who was a member of the team. For each recording he sat at rest on the driving seat for 5 min. Reference respiratory signals were taken simultaneously. Signals were acquired in 2 different series, one from the seat back and the other from the seat belt.

#### 2.4.1. Series 1: Signal Acquisition from the Seat Back

**Figure 10:**
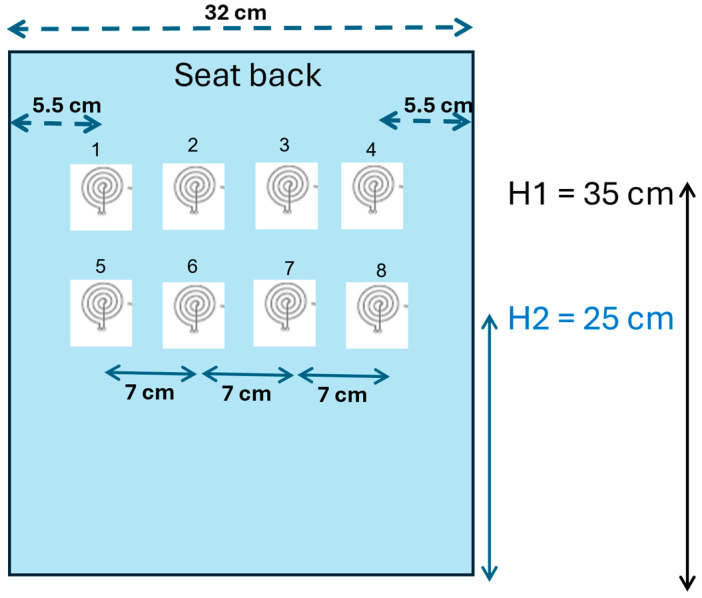
Schematic illustrating signal acquisition on the seat back. Circular electrodes can be placed at up to 8 different positions to acquire respiratory signals, either sequentially or simultaneously.

In this series, inductive respiratory signals at different locations on the back of the subject were measured, as shown in the schematic in Figure 10. Data acquisition was carried out in five steps:

1. One A.73.20 electrode was used to sequentially take a total of 8 recordings at locations #1, #2, #3, #4, #5, #6, #7, and #8.
2. One A.73.20 electrode was folded and used to sequentially take a total of 8 recordings at locations #1, #2, #3, #4, #5, #6, #7, and #8.
3. Three A.73.20 electrodes were used to simultaneously take a total of 3 recordings at locations #1, #2, and #3.
4. Four A.73.20 electrodes were used to simultaneously take a total of 4 recordings at locations #2, #3, #6, and #7.
5. One A.73.20 electrode and one A.73.15 electrode were used to sequentially take a total of 2 recordings at location #3.

The goals of this series are manyfold:

- Assess the signal quality when signals are acquired on the back;
- Test if the signal quality is better when the electrodes are folded;
- Test if the electrode position, namely on the upper back vs. on the lower back, has an influence on the signal quality;
- Test if the signal quality is better when taken from the seat back compared to from the seat belt;

#### 2.4.2. Series 2: Signal Acquisition on the Seat Belt

**Figure 11:**
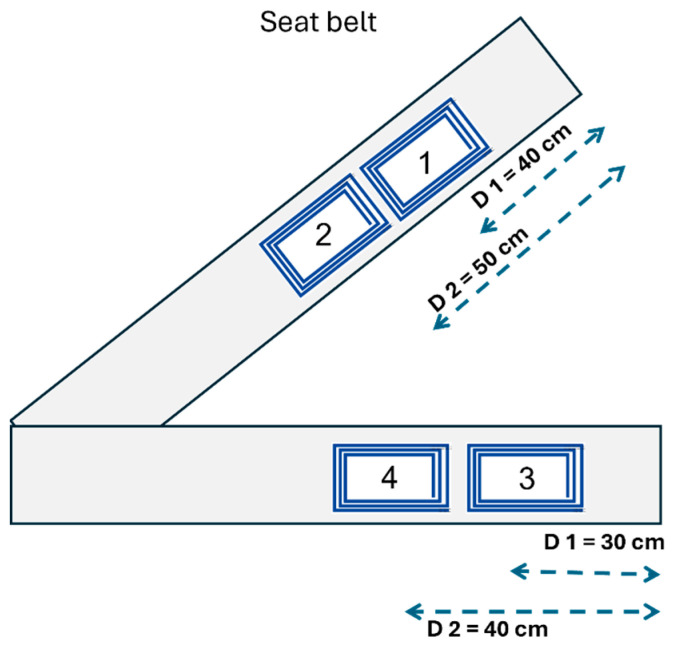
Schematic illustrating signal acquisition on the seat belt. Rectangular or circular electrodes can be placed at up to 4 different positions to acquire respiratory signals, either sequentially or simultaneously.

In this series we measure the inductive respiratory signals at different locations on the chest and the waist of the subject, as illustrated in the schematic in Figure 11. Data acquisition was conducted in four steps:

1. One A.73.39 electrode was used to sequentially take a total of 8 recordings at locations #1, #2, #3, and #4.
2. Four A.73.39 electrodes were used to simultaneously take a total of 4 recordings at locations #1, #2, #3, and #4.
3. Two A.73.20 electrodes were used to simultaneously take a total of 2 recordings at location #3 on the seat back and location #1 on the seat belt.
4. Four electrodes (A.73.12, A.73.20, A.73.39, and A.73.40) were used to sequentially take a total of 4 recordings at location #1.

The goals of this series are also manyfold:

- Assess the signal quality when signals are acquired on the seat belt;
- Test if the electrode position, namely on the chest vs. on the waist, has an influence on the signal quality;
- Test if the signal quality is better when taken on the seat belt compared to on the seat back.

**Figure 12:**
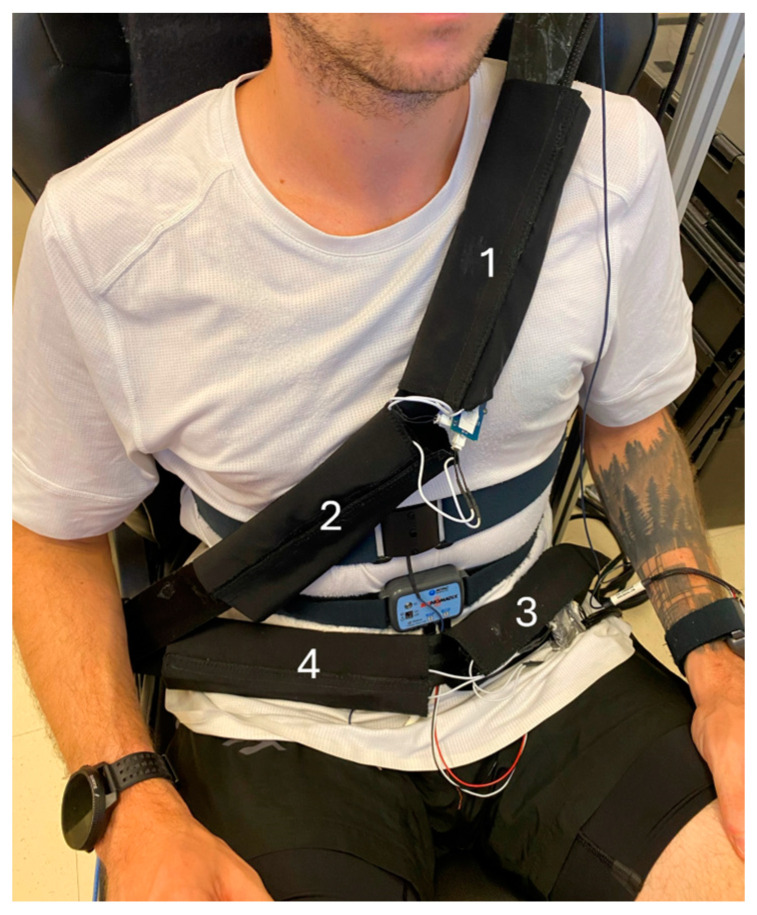
Full electrode integration. Textile inductive electrodes on the seat belt (positions #1, #2, #3, and #4) and the Biopac MP160 respiration strap are shown. Inductive electrodes on the seat back are not visible, hidden by the subject’s torso.

Full electrode integration is shown in Figure 12.

### 2.5. Signal Pre-Processing

Reference respiratory signals were decimated from 1 kHz to 40 Hz to fit the sampling rate of inductive respiratory signals. Glitches at the beginning and end of the recording were excluded from all the reference and inductive signals.

**Figure 13:**
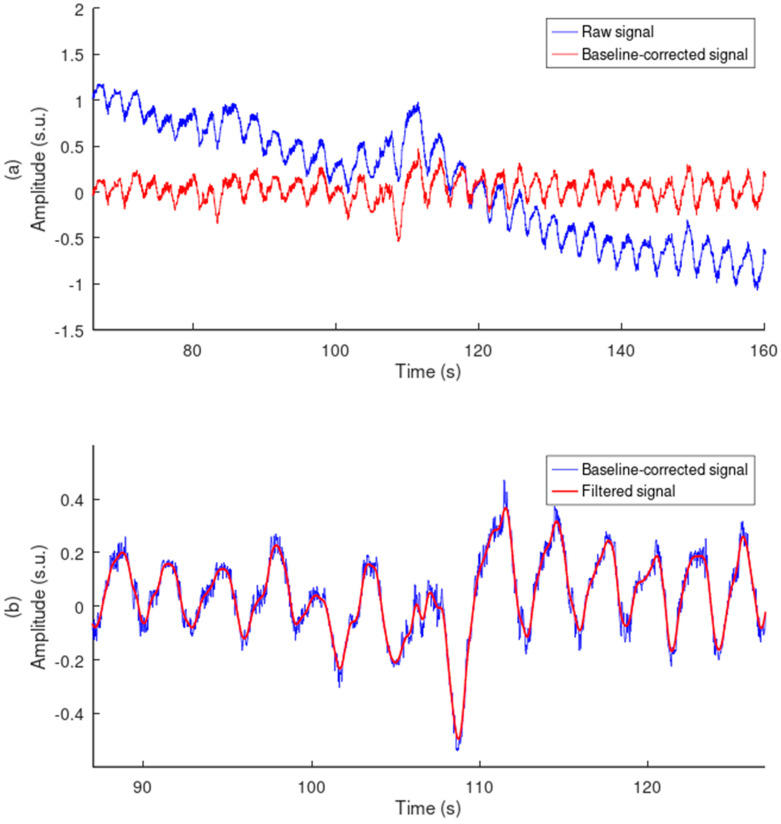
Signal preprocessing. (**a**) Baseline removal example. (**b**) Signal filtering example. Amplitudes are in standard score unit. The signal was acquired with the electrode A7320 positioned flat on the seat back at position #4.

Signal filtering was performed in two steps. Baseline wander was first removed from the raw signal by subtracting a moving average featuring a window of 4 s and an offset of 1 s (Figure 13a). A 20th order FIR lowpass filter of 0.917 Hz cutting frequency was then applied to the baseline-corrected signal in both the forward and reverse directions (Figure 13b). The 0.917 Hz cutting frequency is the upper value of the frequency range [0.066 Hz, 0.917 Hz] that targets a useful range of breathing rate [defined as 4 breaths/min, 55 breaths/min].

### 2.6. Metric to Quantify Assessment of the Signal Quality

To assess the signal quality of the inductive respiratory signal, four signal quality indexes (SQIs) were created.

The first SQI is the signal-to-baseline ratio, SBR:

(2) $$SBR=\frac{Power\;(S_{f})}{Power\;(B_{sl})}$$

The second SQI is the signal-to-high-frequency-noise ratio, SHR:

(3) $$SHR=\frac{Power\;(S_{f})}{Power\;(N_{hf})}$$

The third SQI is the median-to-mean ratio, MMR, calculated as

(4) $$MMR=\frac{median\;(S_{f}^{\prime })}{mean\;(S_{f}^{\prime })}$$

where

(5) $$Power\left(S_{f}\right)=\frac{1}{n}\sum_{i=1}^{n}{S_{f}}_{i}^{2}$$

B_sl_ = S_r_ − S_bc_ (6)

N_hf_ = S_bc_ − S_f_ (7)

and the variables are defined as follows:

- S_r_—raw signal;
- S_bc_—baseline-corrected signal;
- S_f_—filtered signal;
- S_f_’—first derivative of the filtered signal;
- B_sl_—baseline of the signal;
- N_hf_—high-frequency noise;
- N—the number of samples in the signal.

The ratios in Formulas (2)–(4) are calculated in the time domain.

The mean of a population is much more sensitive to outliers than the median. Therefore, MMR is a good reflection of motion artifacts. In the case of respiratory signals, we expect the median to be lower than the mean, with motion artifacts increasing this effect. The lower the MMR, the greater the impact of motion artifacts on the signal. MMR is calculated on the filtered signal to discriminate motion artifacts from baseline wander. The use of the first derivative in the calculation of MMR amplifies the difference between motion artifacts and the rest of the signal.

Baseline wander, motion artifacts, and high-frequency noise are the three most important types of noise [8,9,10] to consider in inductive respiratory signals. SBR, SHR, and MMR were preferred over the classic signal-to-noise ratio (SNR) because they provide more accurate information about the specific contribution of each type of noise. Furthermore, since baseline wander and motion artifacts are the dominant types of noise, SNR would reflect mostly SBR and MMR. Using separate SQIs allows a much more accurate evaluation of high-frequency noise through SHR.

The fourth SQI used In this study is the spectral correlation between the inductive and reference respiratory signals, SPC:

(8) SPC=Correlation(reference spectrum,inductive spectrum)

**Figure 14:**
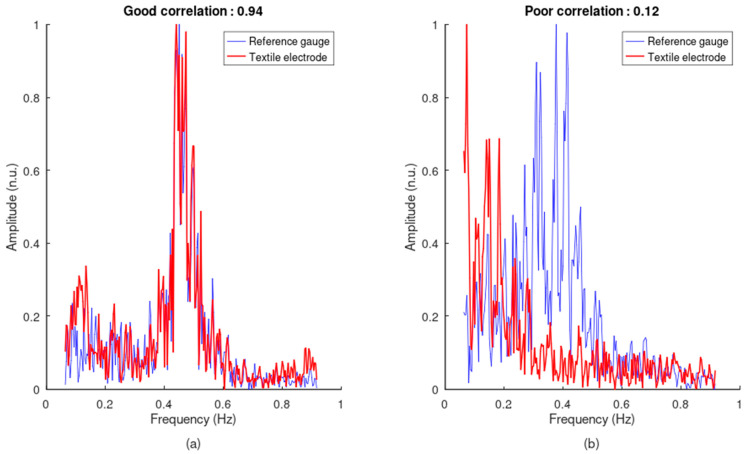
Spectral correlation between the reference and inductive respiratory signals. (**a**) Good spectrum overlap, with a correlation coefficient of 0.94. (**b**) Poor spectrum overlap, with a correlation coefficient of 0.12. All amplitudes are in normalized units.

Normal breathing rate ranges from approximately 4 breaths/min to 55 breaths/min, leading to a targeted frequency range from 0.066 Hz to 0.917 Hz. For each measured inductive signal, fast Fourier transforms of the reference and inductive signals were performed, cropped to the targeted frequency range, and normalized. The correlation between the spectrums of the reference and inductive signals was then calculated. The higher the SPC, the more likely it is to retrieve an accurate breathing rate from the inductive signal. The method is illustrated in Figure 14.

The signal quality metric, SQM, is formulated as follows:

(9) $$SQM=\frac{SBR+SHR+MMR+SPC}{4}$$

Here are the details of the calculations:

1. All recordings from this study are put in a common pool.
2. The value of SBR is calculated for each recording. The set of values of SBR for all recordings is then normalized between zero and one. The process applied to SBR is repeated for SHR, MMR, and SPC.
3. The normalized indexes, SBR, SHR, MMR, and SPC, are averaged into one metric, giving one value for each recording of this study.
4. The set of average values is normalized, again between zero and one, to provide the SQM, with one value for each recording of this study.

Consequently, each signal has a unique score, quantifying its quality.

### 2.7. Ranking Method to Evaluate the Design Configurations

We define the design configuration as a realistic way of integrating the textile inductive electrodes into an automobile’s operational environment to detect respiration. Each configuration indicates the number and position of electrodes on the seat back or the seat belt. In this study, the design configurations are created by pooling the signal recordings in 6 groups, as follows:

1. Upper back flat: recordings from the electrodes flat on the seat back, covering only the upper back (electrodes 1 to 4 in Figure 10).
2. Upper back folded: recordings from the electrodes folded on the seat back, covering only the upper back (electrodes 1 to 4 in Figure 10).
3. Lower back flat: recordings from the electrodes flat on the seat back, covering only the lower back (electrodes 5 to 8 in Figure 10).
4. Lower back folded: recordings from the electrodes folded on the seat back, covering only the lower back (electrodes 5 to 8 in Figure 10).
5. Belt on chest: recordings from the electrodes on the belt, covering only the chest (electrodes 1 and 2 in Figure 11).
6. Belt on waist: recordings from the electrodes on the belt, covering only the waist (electrodes 3 and 4 in Figure 11).

The goal of the ranking method is to allow a direct comparison between the design configurations instead of between the individual electrodes. All signals in the study were ranked from the best to the worst and dispatched into the 6 groups defined above. The design configurations were then ranked as a function of the number of recordings with the higher ranked signals they contain.

The first step of the ranking method is to transform the set of SQM scores into a set of SRM scores (signal ranking metric). To do so, the set of SQM values is sorted into descending order—the best signal has a score of 1 and the worst a score of 0. For N recordings in the study, the best signal receives a score of N, the second best a score of N − 1, and so on, until a score of 1 is given to the worst quality signal. Then the set $\left\{N,N-1,\ldots ,1\right\}$ is normalized between zero and one to provide the SRM—the signal with the highest rank has a score of 1 and that with the lowest rank a score of 0:

(10) $$SRM=normalize(\left\{N,N-1,\ldots ,1\right\})$$

The second step is to dispatch the SRM scores into the 6 groups previously stated. The ranking method compares the 6 best signals of each group to allocate the same number of samples in each group, for a total of 36 analyzed signals.

## 3. Results

### 3.1. Verification of the Signal Ranking Metric

**Figure 15:**
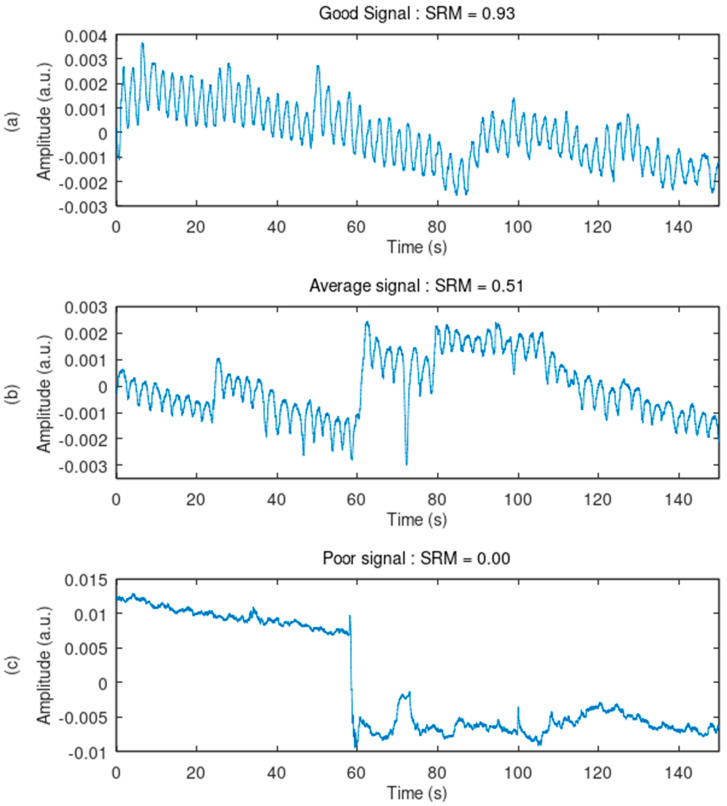
Ranking of the signals based on quality: illustration of the method. Three signals, with high, average, and poor signal quality rankings, are displayed. (**a**) The good signal can allow peak detection for breathing rate calculation. (**b**) The average signal can also provide breathing rate but is challenged by baseline wander, light motion artifacts, and light high-frequency noise. (**c**) The poor signal does not allow breathing rate detection. It suffers from high-amplitude baseline wander, severe motion artifacts, and serious high-frequency noise.

**Figure 16:**
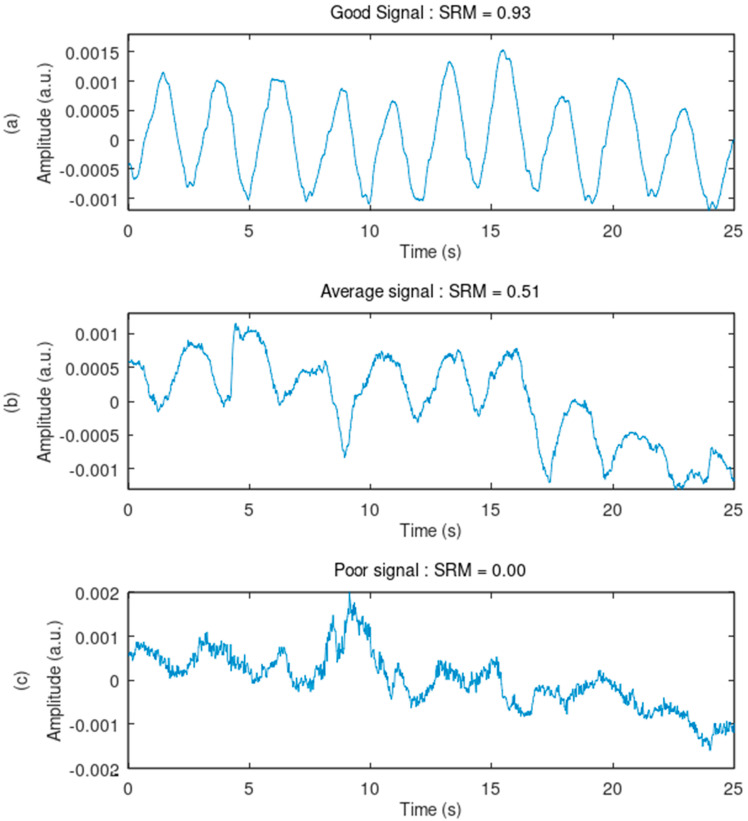
Ranking of the signals based on quality. These plots provide a closer look at the signals from Figure 15 and confirm the conclusions. (**a**–**c**) The presence of high-frequency noise increases with a decreasing signal quality ranking. The ability to allow peak detection for breathing rate calculation decreases with a decreasing signal quality ranking.

The SRM score is effective at ranking the signal quality of the recordings from good to poor. All the ranked signals were displayed and visually inspected. Decreasing SRM scores displayed signals with decreasing quality, i.e., with higher baseline wander, more high-frequency noise, higher motion artifact, and a lesser capacity to allow the calculation of breathing rate through peak detection. Figure 15 and Figure 16 illustrate three ranked samples. Verification of the individual SQIs is detailed in Appendix A.

### 3.2. General Assessment of the Signal Quality

**Figure 17:**
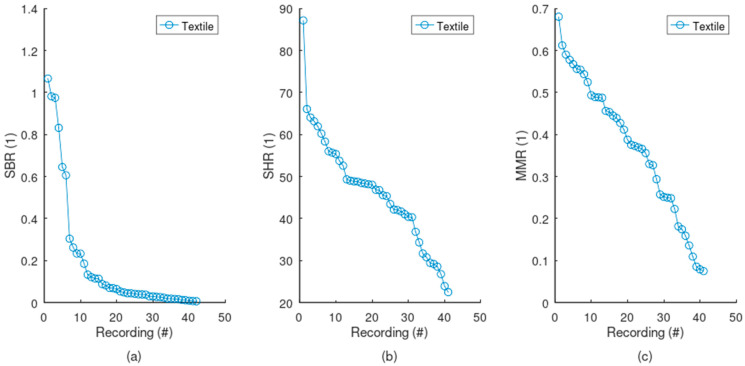
Noise profile from the textile electrodes. The SQIs for all the recordings are sorted in decreasing order and plotted. (**a**) A SBR below 1 indicates that the baseline wander usually has higher power compared to the signal. Baseline wander is therefore a significant contributor to the noise. (**b**) On the contrary, a high SHR indicates that the high-frequency noise typically has much lower power than the signal and therefore is not an important noise. (**c**) According to visual inspection of the whole set of signals, motion artifact starts to become severe at a very low level of MMR, generally below 0.2.

**Figure 18:**
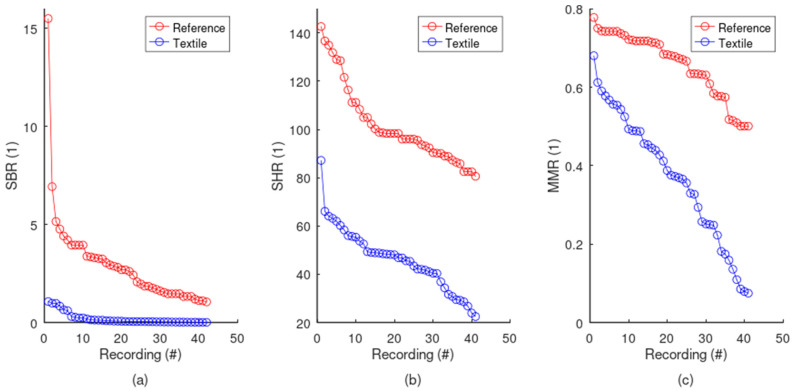
Comparative analysis of the noise profiles: reference vs. textile. The SQIs for all recordings are sorted in decreasing order and plotted for the Biopac reference and the textile electrodes. The reference always displays a higher ratio, resulting in the following: (**a**) less baseline wander, (**b**) less high-frequency noise, and (**c**) less spikes due to motion artifacts.

Noises were thoroughly profiled. The textile electrodes are challenged with serious baseline wander, since the SBR is usually far below 1, i.e., the signal usually has lower power compared to the baseline (Figure 17a). On the contrary, textile electrodes generally display very low levels of high-frequency noise, with a SHR of much higher than 1 (Figure 17b). MMR can only really be appreciated when compared to the reference signal (see Figure 18). Since the reference has almost no motion artifacts, the MMR starts to become challenging below the reference’s minimum MMR value, i.e., below 0.5. Therefore, most of the textile electrodes display some level of motion artifact, but severe cases were observed only at very low MMR values, generally below 0.2. Finally, the signal quality of the textile electrodes is generally lower compared to the reference (Figure 18), with more baseline wander, a higher level of high-frequency noise, and more motion artifacts.

### 3.3. Evaluation of the Design Configurations

**Figure 19:**
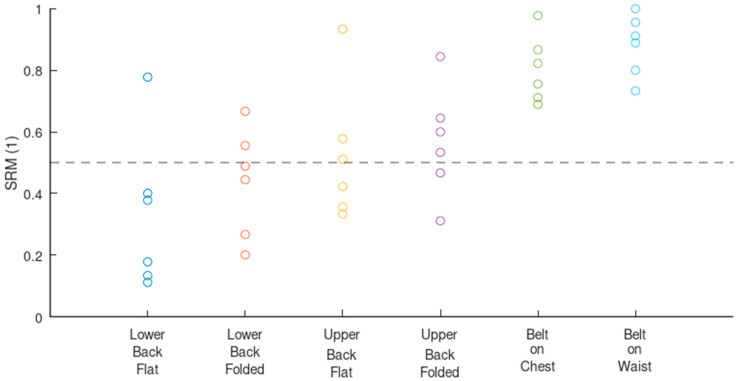
Scatter plot of SRM values for several design configurations on the seat back and the seat belt.

The scatter plot in Figure 19 gives a general overview of how the design configurations are ranked compared to each other. The seat belt displays higher SRM scores spread across a smaller range compared to the seat back. The SRM scores of the seat back are distributed both above and below 0.5, meaning that individual design configurations on the back provided both good and poor signals.

Table 2 quantifies the findings from Figure 19. Growing median and mean SRM values indicate a growing ranking of the following design configurations: lower back flat, lower back folded, upper back flat, upper back folded, belt on chest, and belt on waist. The seat belt ranks higher than the seat back in general, with the range and standard deviations also lower. The upper back outranks the lower back. Folding the electrodes increases the ranking for both the upper and lower back compared to the flat electrodes. Furthermore, belt on waist ranks higher than belt on chest, and provides the highest performance of all the design rankings.

Table 3 details the distribution of the SRM values and confirms the ranking conclusions stated earlier from Table 2, but with a closer look. Generally, the seat back is poorly represented for SRM values over 0.6 compared to the seat belt, which explains the generally higher ranking of the seat belt. The upper back is better represented for a SRM ≥ 0.5 compared to the lower back. Folding the electrodes improves the distribution for both the upper and the lower back when the SRM ≥ 0.5. Finally, where SRM ≥ 0.8, belt on waist takes the lead over belt on chest.

### 3.4. Evaluation of Form Factors and Inter-Electrode Interference

**Figure 20:**
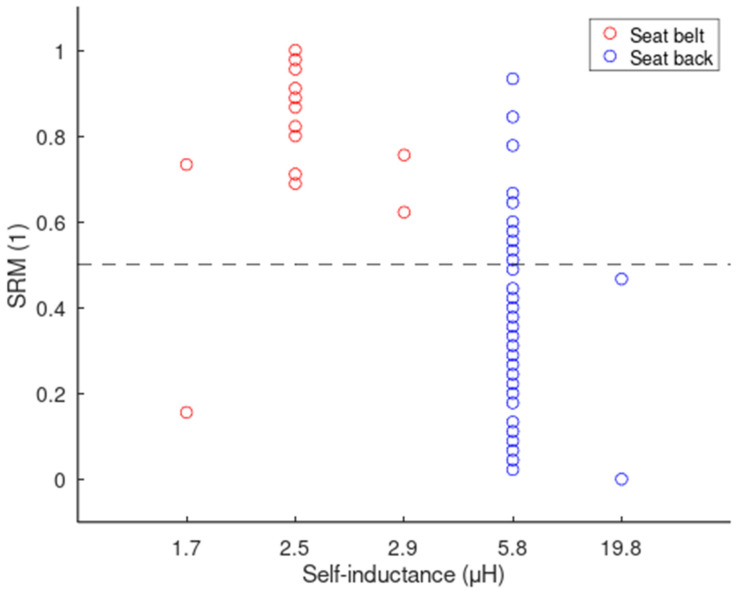
Scatter plot of the SRM values as a function of the electrodes’ self-inductance.

Electrodes had different form factors across different design configurations. In this section, we compare the impact of size and shape on the signal quality compared to the design configurations. According to Table 1, each electrode size or shape had a specific self-inductance, which therefore can be used as a unique surrogate variable for form factors in our data analysis. The SRM scores plotted against self-inductance did not display any trend, as shown in Figure 20. For each self-inductance, the values of SRM are distributed over a wide range. However, when self-inductances are separated into seat back vs. seat belt groups, the previous patterns emerge, with the seat belt having better scores and displaying less variability compared to the seat back. We therefore conclude that design configurations had more of an effect on the signal quality than the form factors.

**Figure 21:**
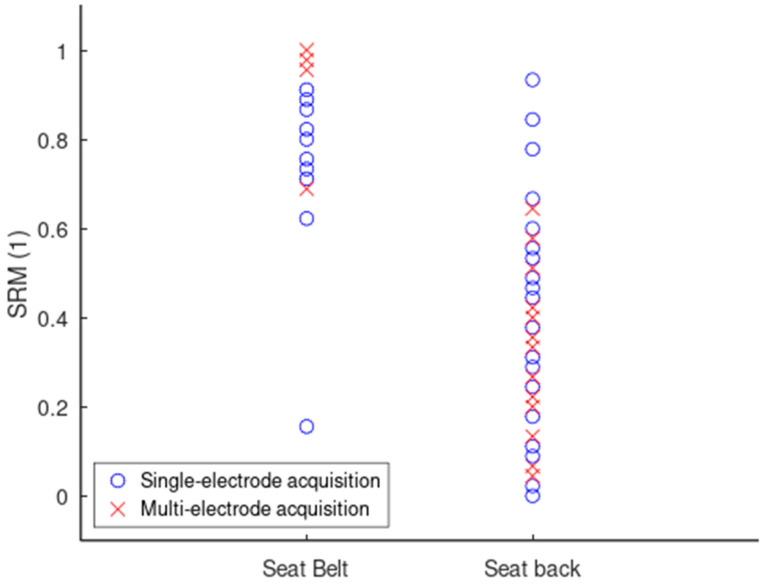
Scatter plot of the SRM values for recordings with single- and multi-electrode acquisition.

Similarly, signal quality was primarily based on where the signals were captured and not on the fact that acquisition was simultaneous. Figure 21 shows that, for a given design configuration, recordings with multi-electrode acquisition have a similar ranking compared to their single-electrode acquisition counterparts. We therefore conclude that design configurations had more of an effect on signal quality compared to the inter-electrode interference.

### 3.5. Basic Design Rules

Evaluation of the design configurations allowed the formulation of three basic design rules.

Rule #1: use a multi-electrode acquisition approach.

It may be difficult to find the ultimate electrode’s location that fits all automobiles’ operational environments, subject sizes, and driving body posture habits. The seat belt is likely a good candidate for electrode placement in general, but its SRM scores are spread over a range of up to 0.29 in this study. This means there could be a loss of ranking of up to 29% if only one electrode was used on the seat belt. This variability issue can be improved by using several electrodes and picking the signal with the best score. Furthermore, the seat back cannot necessarily be ruled out in case of seat belt failure, due, for example, to the subject’s driving habits. The most robust system will contain electrodes in both the seat back and the seat belt.

Rule #2: place the electrodes in locations that maximize breathing-induced body displacement.

Locations in which to integrate breathing electrodes are not equal in their ability to provide a good quality signal. In Figure 19, Table 2 and Table 3 clearly demonstrate that signal quality is proportional to the breathing-induced body displacement. As a matter of fact, anatomically, there is much more breathing-induced movement where the seat belt covers the waist compared to where the seat back covers the lower back. Similarly, a seat belt covering the chest anatomically provides more breathing-induced movement compared to a seat back covering the upper back.

Rule #3: use mechanical amplifying methods in locations with little potential for breathing-induced displacement.

In anatomical places where the breathing-led movement may be low, a mechanical amplifying method like a folded electrode could be an effective solution (see Figure 19, Table 2 and Table 3).

## 4. Discussion and Conclusions

The goal of this feasibility study was to introduce a quantitative method based on signal quality to guide the integration of textile inductive electrodes in automotive applications. To meet that goal, we performed the following:

- built a breathing sensing system with textile inductive electrodes, dedicated circuitry, and acquisition software
- assembled several realistic design configurations, by integrating various numbers of electrodes in various places on the seat back or seat belt
- created a metric to quantitatively assess the signal quality of inductive respiratory signals
- created and used a ranking method based on signal quality to evaluate the design configurations
- deduced basic design rules from the evaluation of the design configurations
- To our knowledge, the present study is the first to evaluate design configurations with a quantitative assessment of signal quality. A rationale has been given for the number and position of electrodes on either the seat back or seat belt. The quantified evaluation approach also promotes systematic and educated choices during the design process. The ranking method has the advantage of being versatile, accommodating any number of samples and any type of design configuration.

The study is also the first to propose design rules targeting the integration of textile inductive electrodes for respiratory monitoring in automotive applications. It is indeed possible to gather respiratory signals of optimal quality, under one assumption and one condition. Firstly, the assumption is that “good quality” strictly means “allowing the calculation of breathing rate through peak detection”. Tracking more complex variables, like respiratory volumes for example, may require higher quality and precise calibration that are out of the scope of this study. We deliberately focused on breathing rate because one of the main purposes of recording respiratory signals in automobiles is to extract the breathing rate, which could be used by algorithms to detect events such as drowsiness [20,28,29,30]. Secondly, the condition is to follow three design rules: (a) use a multi-electrode acquisition approach, (b) place the electrodes in locations that maximize breathing-induced body displacement, and (c) use mechanical amplifying methods such as folding the electrodes in locations with little potential for breathing-induced displacement.

The sensing principle explored in this study involves the measurement of breathing-induced body displacements with the electrode’s inductance variations. The physical phenomena involved were not the main focus of this study, but previous works proposed two possible explanations. The first one is based on the effect of the subject’s conductive body moving within the time-varying magnetic field of the textile inductive electrode. Eddy currents within the body generate a secondary magnetic field that opposes the electrode’s primary field, thus reducing the inductance, L_e_ [8,9]. The second explanation is based on the geometrical properties of the textile inductive electrode itself. Breathing-induced bending or folding of the electrode changes the distance between the wires of the coil, which influences the local mutual inductance between them, and therefore changes the total value of inductance, L_e_ [27,31]. Further investigations are required to determine which of the two physical phenomena was the most involved in the present study.

Our system places flexible electrodes directly on the surface of the seat back or the seat belt. Beyond making the technology seamless for the user, this brings several advantages compared to other inductive systems. For example, a shorter distance between the body and the electrode may improve signal quality, as suggested by another study that put the coil inside the seat [9]. Flexible electrodes may also enhance the interaction between the body and the electrode and therefore increase the signal quality compared to other, stiff settings [8,10].

For this study, we deliberately chose a static setting, where the subject just sits still, versus a dynamic one where he would be performing a driving movement. This allowed us to isolate the key variable of the study, i.e., the sensor position, either on the back or on the belt. This choice does not affect the realism of the results since breathing rate calculation can only be done on a low-noise signal, even if it means filtering it before analysis. The main difference between static and dynamic settings is motion artifacts. A study in a dynamic setting could identify the electrode positions that are more prone to motion artifacts and then predict the availability of good quality signals for each position.

Finally, we did not place stress on other design parameters like size and shape since no major trend was discovered compared to the effects of the design configurations. However, they are worth studying at extreme values to understand how the system would perform in edge cases. A few examples are as follows: What could be the impact of large and unconventional geometries on the seat back? Can we use electrodes of very different inductances in the same design configuration? If one electrode lets in a lot of 60 Hz interference, what would be the impact on the other electrodes?

## Figures and Tables

**Table 1:** Characteristics of each type of electrode. Inductances were measured by an Instek LCR-916 Handheld LCR Meter at 100 kHz.

Prototype	Outer Diameter (mm)/Area	Inner Diameter (mm)/Area	Number of Turns	Space Between Turn (mm)	Measured Inductance (μH)
A.73.12	60	0	10	1.5	2.9
A.73.15	120	0	20	1.5	19.8
A.73.20	80	5	13	1.5	5.8
A.73.39	150 × 50	115 × 10	5	3	2.5
A.73.40	100 × 50	60 × 10	5	3	1.7

**Table 2:** Statistics for the SRM values.

	Lower Back Flat	Lower Back Folded	Upper Back Flat	Upper Back Folded	Belt on Chest	Belt On Waist
Median	0.28	0.47	0.47	0.57	0.79	0.90
Mean	0.33	0.44	0.52	0.57	0.80	0.88
STD	0.25	0.18	0.22	0.18	0.11	0.10
Range	0.67	0.47	0.60	0.53	0.29	0.27

**Table 3:** Distribution of the SRM values.

	Lower Back Flat	Lower Back Folded	Upper Back Flat	Upper Back Folded	Belt on Chest	Belt on Waist
SRM ≥ 0.8	0	0	1	1	3	5
SRM ≥ 0.7	1	0	1	1	5	6
SRM ≥ 0.6	1	1	1	3	6	7
SRM ≥ 0.5	1	2	3	4	6	7
SRM ≥ 0.4	2	4	4	5	6	7

**Figure A1:**
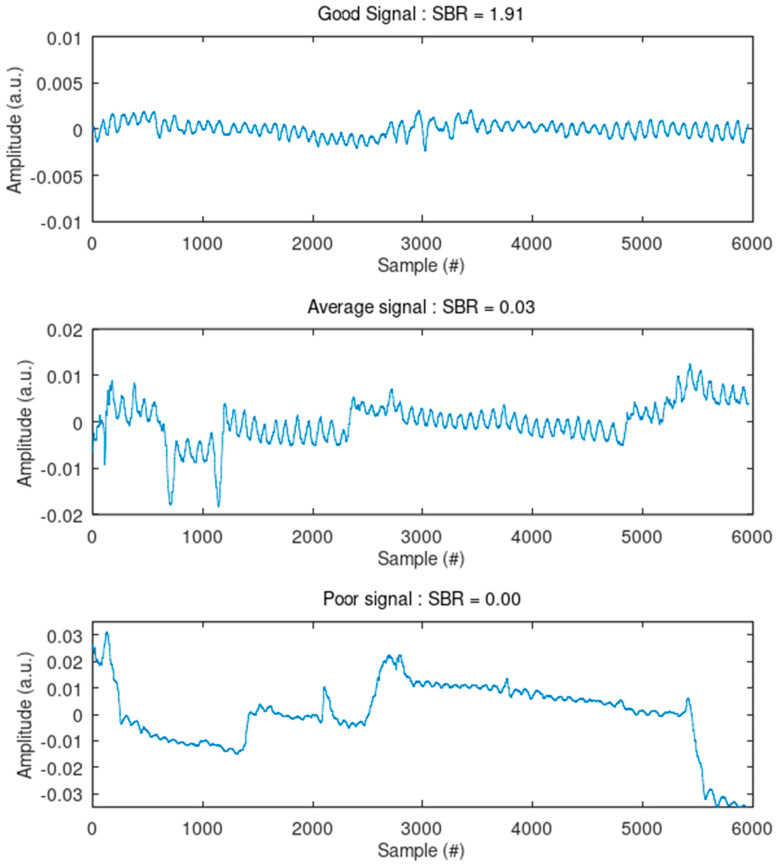
Verification of the signal-to-baseline ratio, SBR. Baseline wander is inversely proportional to the SBR, as expected.

**Figure A2:**
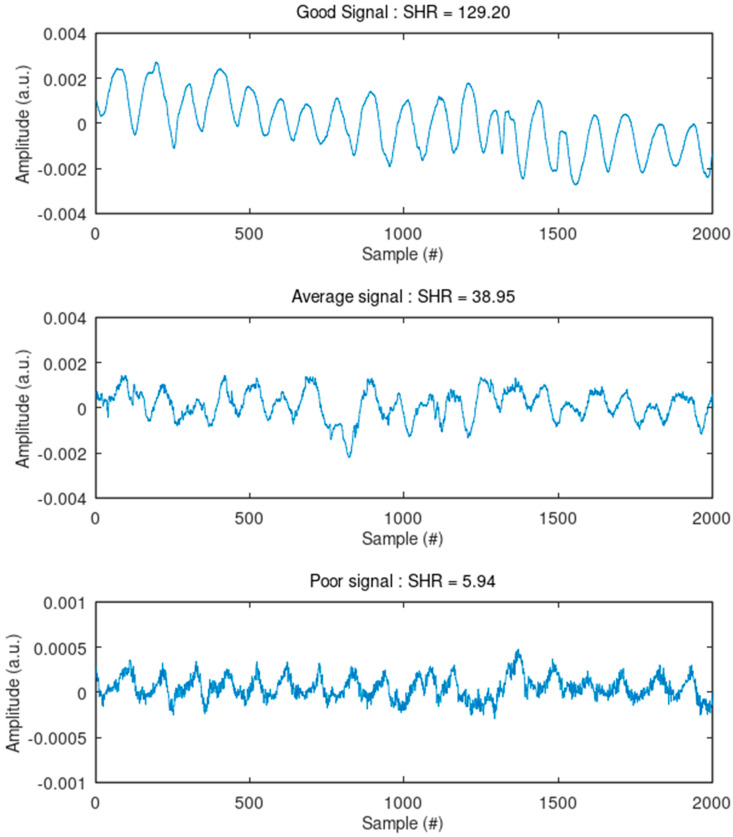
Verification of the signal-to-high-frequency-noise ratio, SHR. The presence of high-frequency noise is inversely proportional to the SHR, as expected.

**Figure A3:**
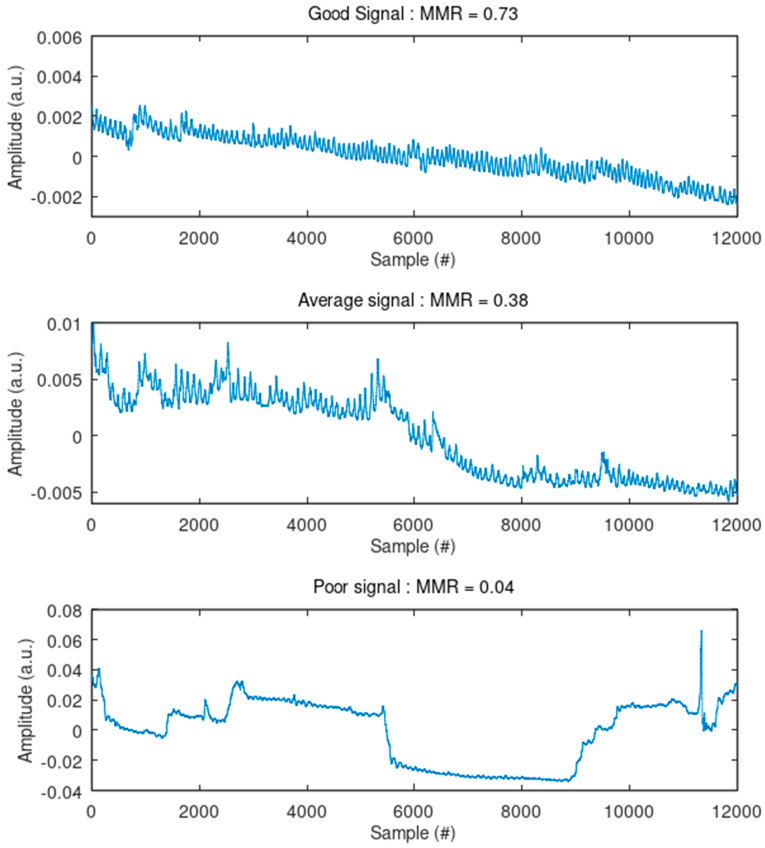
Verification of the median-to-mean ratio, MMR. The presence and amplitude of motion artifacts are inversely proportional to the MMR, as expected.

**Figure A4:**
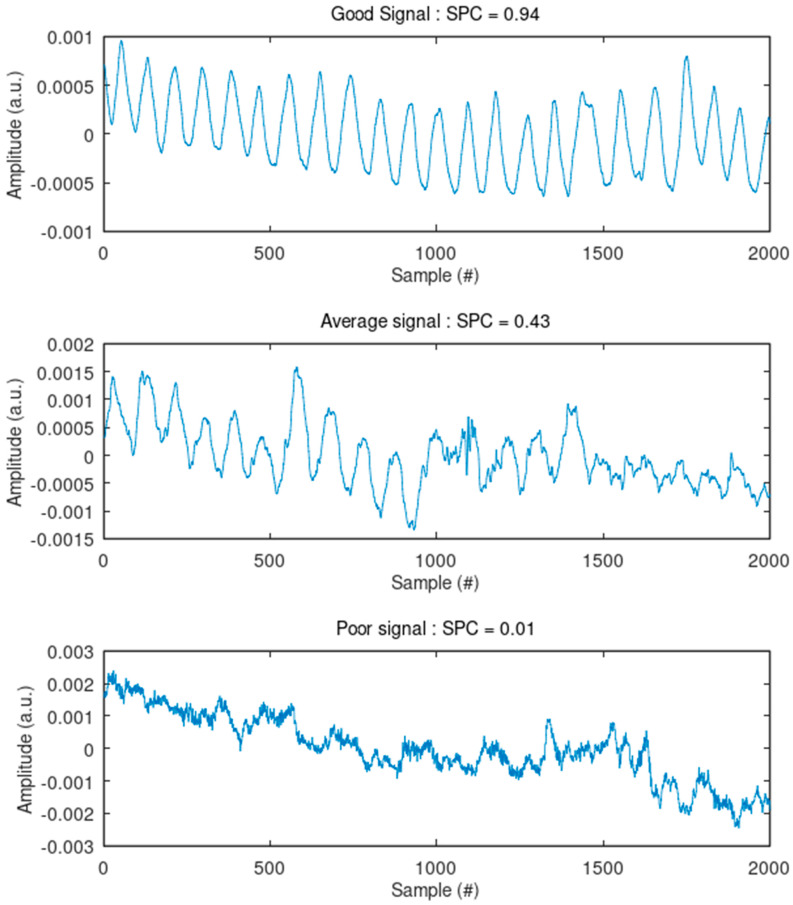
Verification of the spectral correlation, SPC. The presence of a dominant frequency is inversely proportional to the SPC, as expected.

## Data Availability

The data for the project were collected at the electrical engineering department, École de Technologie Supérieure. For ethical and confidentiality reasons, the authors cannot provide public access to them. Nevertheless, the authors agree to make data and materials supporting the results or analyses available for the investigation of scientific integrity if necessary.

## Appendix A. Verification of the Signal Quality Indexes

This appendix presents signal samples with decreasing values of the signal quality indexes (SQIs). All examples demonstrated decreasing quality for decreasing SQIs. We therefore conclude that the SQIs are performing as intended.
